# Discovery of Phylloseptins that Defense against Gram-Positive Bacteria and Inhibit the Proliferation of the Non-Small Cell Lung Cancer Cell Line, from the Skin Secretions of *Phyllomedusa* Frogs

**DOI:** 10.3390/molecules22091428

**Published:** 2017-08-29

**Authors:** Jia Liu, Qing Wu, Lei Li, Xinping Xi, Di Wu, Mei Zhou, Tianbao Chen, Chris Shaw, Lei Wang

**Affiliations:** 1 School of Chinese Materia Medica, Beijing University of Chinese Medicine, Beijing 102488, China; jliu19@qub.ac.uk; 2 Natural Drug Discovery Group, School of Pharmacy, Queen’s University, Belfast BT9 7BL, UK; X.Xi@qub.ac.uk (X.X.); dwu03@qub.ac.uk (D.W.); m.zhou@qub.ac.uk (M.Z.); t.chen@qub.ac.uk (T.C.); Chris.Shaw@qub.ac.uk (C.S.); l.wang@qub.ac.uk (L.W.)

**Keywords:** phylloseptins, antimicrobial peptides, anti-biofilm, cytostatic

## Abstract

The growing occurrence of bacterial resistance to conventional antibiotics has called for the development of new classes of antimicrobial agents. Antimicrobial peptides (AMPs) with broad antimicrobial spectrum derived from frog skin secretions have been demonstrated to be promising candidates for new antibiotic development. A proven rich source of these compounds are the skin secretions of the frogs in the *Phyllomedusa* genus. In this study, two novel phylloseptin peptides—phylloseptin-PTa and phylloseptin-PHa—were isolated from the skin secretions of the South American frogs, *Phyllomedusa tarsius* (*P. tarsius*) and *Phyllomedusa hypochondrialis* (*P. hypochondrialis*) through parallel transcriptomic and peptidomic studies. Replicates obtained by chemical synthesis were structurally analysed and shown to adopt an α-helix configuration in an amphiphilic environment. Both peptides demonstrated antimicrobial activities against planktonic Gram-positive bacteria strains, including *Staphylococcus aureus*, *Enterococcus faecalis* and methicillin-resistant *Staphylococcus aureus* , biofilms, as well as cytostatic effects on the non-small cell lung cancer cell line, NCI-H157, with relatively low haemolysis on horse erythrocytes and low cytotoxicity on the human microvascular endothelial cell line, HMEC-1. The discovery of phylloseptin peptides may further inspire the development of new types of antibiotics.

## 1. Introduction

Decades of research has revealed amphibian skin secretions as great natural sources of bioactive compounds, such as peptides, steroids, alkaloids and biogenic amines [1]. Among the bioactive peptides, antimicrobial peptides (AMPs) comprise the group with the most immediate therapeutic potential owing to their antimicrobial superiority over conventional antibiotics. The AMPs derived from amphibian skin secretions have remarkable activities against a great range of microorganisms, falling into the categories of bacteria, fungi, protozoa and even viruses [2,3]. It has been widely accepted that cationic AMPs initially interact with the anionic phospholipids in the cell membrane by electrostatic forces, followed by destruction of cell membranes via hydrophobic interactions through several different mechanisms and ultimately lead to cell death [4,5]. Thus, AMPs with these distinctive membrane interactions may represent a powerful weapon in the fight against resistant pathogenic microorganisms.

The skin secretions of neotropical frogs of the subfamily Phyllomedusinae, which have attracted research attention worldwide in recent decades, contain an abundant variety of active peptides [6] making these secretions an excellent source for AMP discovery [7]. This subfamily is further classified into seven major genera, namely *Agalychnis*, *Cruziohyla*, *Hylomantis*, *Pachymedusa*, *Phasmahyla*, *Phrynomedusa*, and *Phyllomedusa*, in which diverse bioactive peptides have been found and grouped into structurally-related families [8]. The high degree of conservation present in the coding sequence of the signal peptides in their biosynthetic precursors suggests their homologous phylogenetic ancestry, while the high degree of variation displayed in the mature peptides reveals their natural molecular evolutionary strategies in different environments [9,10,11].

Phylloseptin peptides, representing a class of AMPs discovered from the skin secretions of Phyllomedusinae subfamily leaf frogs, share several common primary structure features, including a general length of 19–21 amino acid residues, an highly-conserved N-terminal FLSL{I/L}P sequence, C-terminal amidation and α-helical configurations in membrane or membrane-mimetic environments [12]. While the C-terminal amidation is a common post-translational modification found in many AMPs, the action of the amidation varies, for example, enhancing the helical stability of the peptides on membrane surface, or increasing the cationicity of peptides, which improves their antimicrobial activities [13,14].

Due to their potent antimicrobial activities and relatively low haemolytic effects, phylloseptin peptides are considered to be promising antimicrobial agents [15,16,17]. They have been reported to have broad-spectrum antimicrobial activities against bacteria in both planktonic and biofilm growth modes, fungi and protozoa while possessing low haemolytic activities [16,17,18,19]. Additionally, anticancer activities were revealed in a phylloseptin peptide, phylloseptin-PBa, in a recent study, where the peptide selectively interacted with pathogens and cancer cells, and showed lower cytotoxicity against normal mammalian cells [15]. In addition, several phylloseptin peptides have been found to be effective against the viability of protozoa such as *Trypanosoma cruzi* (*T. cruzi*) and *Leishmania*, an example of which being phylloseptin-7 and phylloseptin-8, isolated by Pinto and his colleagues from *Phyllomedusa nordestina*. These peptides were able to target the plasma membranes of *T. cruzi* and lead to cell death, which make them promising anti-parasitic drug candidates [20].

In this study, two phylloseptin peptides—phylloseptin-PTa and phylloseptin-PHa—were individually discovered from the skin secretions of the Brown-bellied Leaf Frog, *Phyllomedusa tarsius* (*P. tarsius*) and the Orange-legged Leaf Frog, *Phyllomedusa hypochondrialis* (*P. hypochondrialis*), via the employment of ‘shotgun’ cloning of skin secretion-derived cDNAs combined with tandem mass (MS/MS) spectrometric analysis of skin secretion peptides. Replicates of phylloseptin-PTa and phylloseptin-PHa were acquired by solid-phase peptide synthesis, and their biological activities were investigated in various bioassays.

## 2. Results

### 2.1. ‘Shotgun’ Cloning of cDNAs Encoding the Phylloseptin-PTa Precursor from the Skin Secretions of P. tarsius and the Phylloseptin-PHa Precursor from the Skin Secretions of P. hypochondrialis

**Figure 1:**
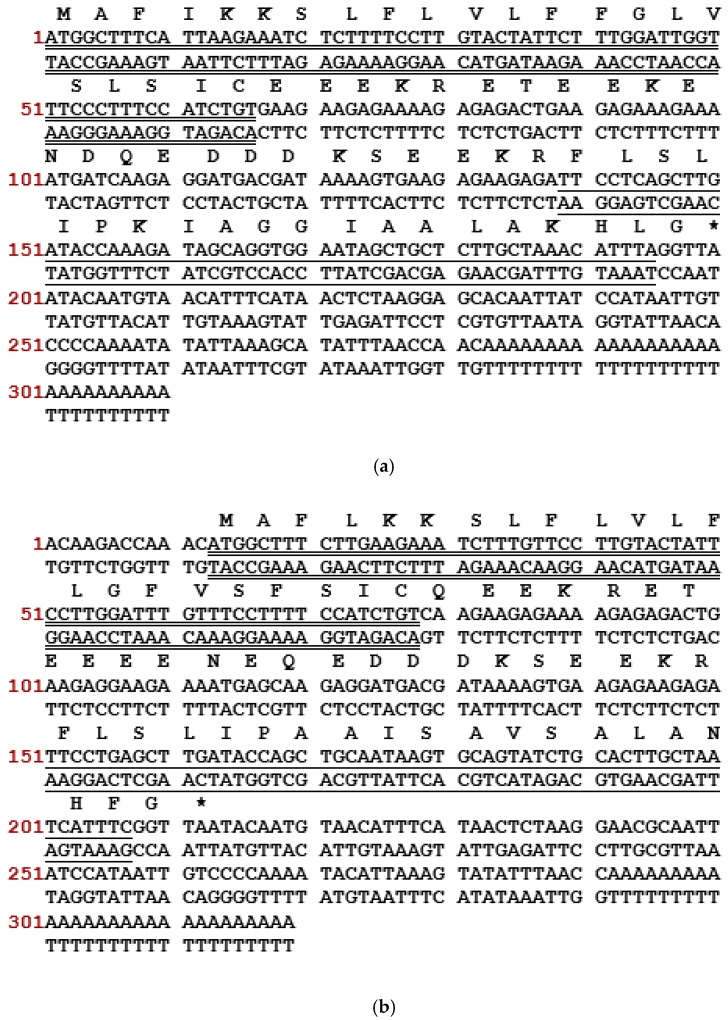
Nucleotide and translated open-reading frame amino acid sequence of cloned cDNA encoding the precursor of (**a**) phylloseptin-PTa and (**b**) phylloseptin-PHa. The putative signal peptide is double-underlined, the mature peptide is single-underlined and the termination codon is marked by an asterisk.

**Figure 2:**
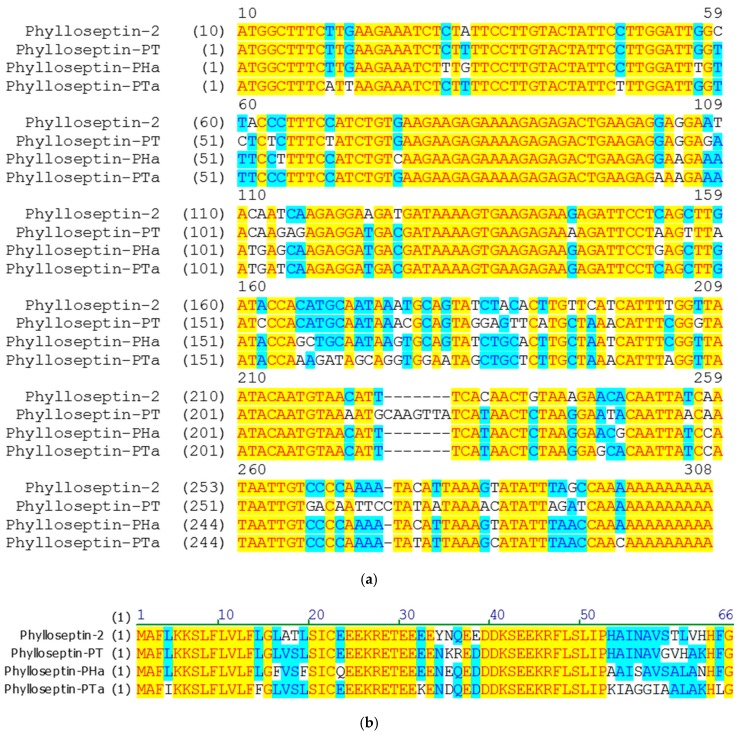
Alignments of (**a**) nucleotides and (**b**) translated open-reading frame amino acids sequences of biosynthetic precursors of phylloseptin-PTa, phylloseptin-PHa, phylloseptin-2 and phylloseptin-PT established by the Vector NTI Advance 11.5.1 package. The identical and conservative regions are highlighted in yellow and blue, respectively.

Through a “shotgun” cloning strategy, the cDNAs encoding biosynthetic precursors of phylloseptin-PTa and phylloseptin-PHa were consistently and repeatedly cloned from the cDNA libraries constructed using the skin secretions of *P. tarsius* and *P. hypochondrialis*, respectively (Figure 1). Both prepropeptides consist of 66 amino acid residues organised in five domains: a putative signal peptide region of 22 amino acid residues, an acidic spacer domain, a typical cleavage site -Lys-Arg- (-KR-), a mature peptide region of 19 amino acid residues and a C-terminal Gly (G) residue which acts as an amide donor for post-translational amidation. BLAST search using the National Center for Biotechnology Information (NCBI) protein Basic Local Alignment Search Tool (BLASTp) showed that the nucleotide and amino acid sequences of both precursors exhibited high degree of similarity to phylloseptin-PT previously identified from *P. tarsius* (Accession LT591888.1) [21], and phylloseptin-2 from *P. hypochondrialis* (Accession AM229009.1) [22] (Figure 2). The prepropeptides-encoding cDNAs sequences of phylloseptin-PTa and phylloseptin-PHa have been deposited in the EMBL Nucleotide Sequence Database under the accession codes LT703448 and LT860204, respectively.

### 2.2. Isolation and Primary Structural Identification of Phylloseptin-PTa and Phylloseptin-PHa

**Figure 3:**
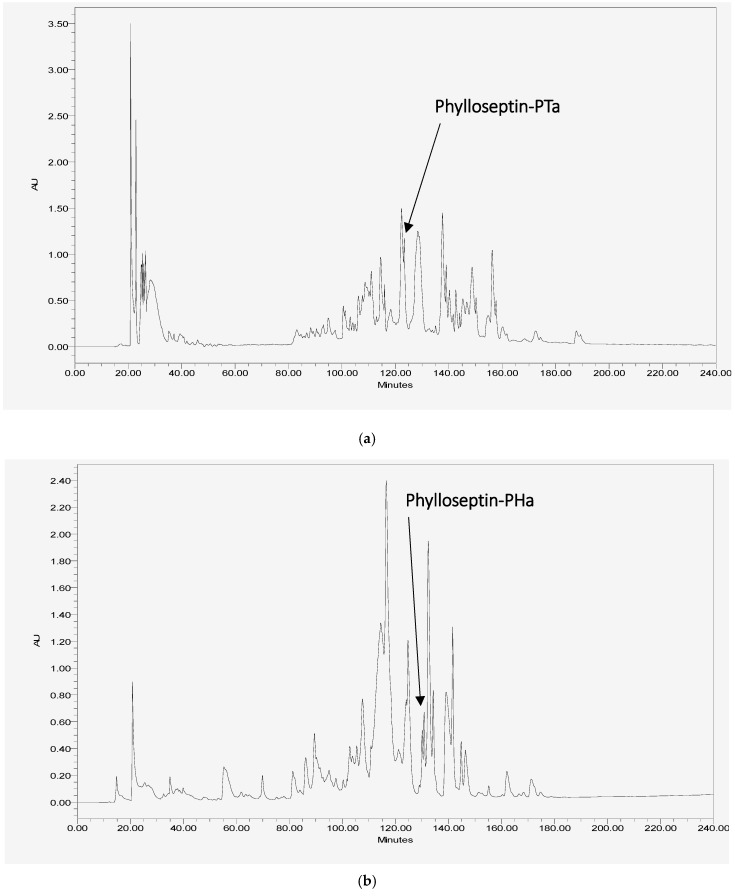
Reversed-phase high performance liquid chromatography (RP-HPLC) chromatograms of the skin secretions from (**a**) *P. tarsius* and (**b**) *P. hypochondrialis* with arrows showing the absorbance peaks corresponding to mature phylloseptin-PTa and phylloseptin-PHa, respectively. The *Y*-axis shows the relative absorbance in absorbance units at a wavelength of 214 nm and the *X*-axis shows the retention time in minutes.

**Figure 4:**
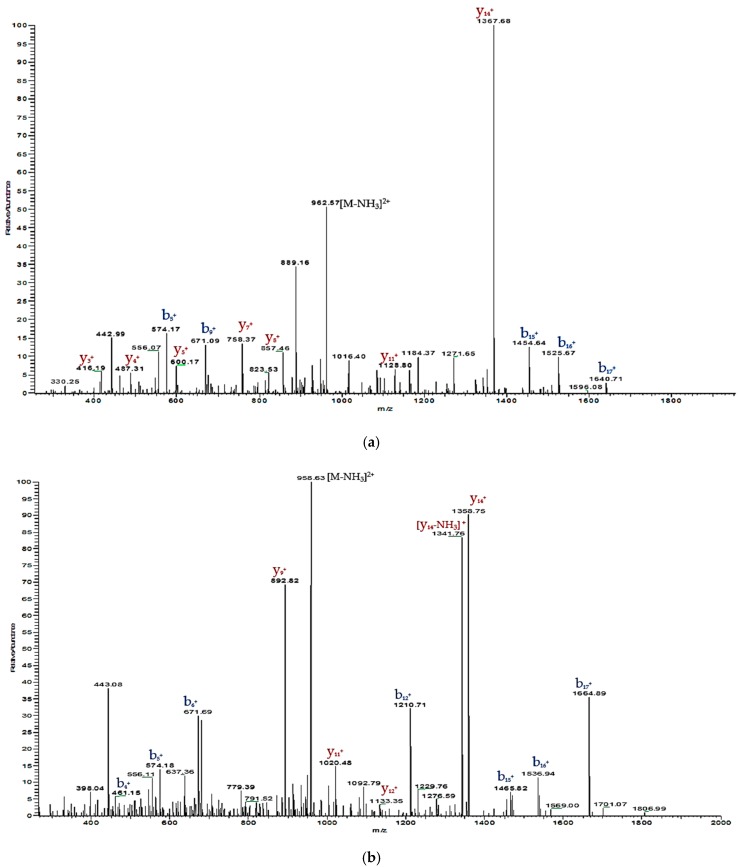
Annotated tandem mass (MS/MS) fragmentation spectra of (**a**) phylloseptin-PTa and (**b**) phylloseptin-PHa with observed *b* ions and *y* ions labelled in blue and red, respectively.

The RP-HPLC fractions containing the peptides, of which the molecular mass obtained by matrix-assisted laser desorption/ionization time-of-flight mass spectrometry (MALDI-TOF MS) were consistent with the computed masses of deduced phylloseptin-PTa and phylloseptin-PHa were further analysed by MS/MS spectrometry (Figure 3 and Figure 4). The fragmented *b* and *y* ions confirmed the primary structures, as well as the post-translational modification of C-terminal amidation of both novel phylloseptins (Table 1).

### 2.3. Verification of Synthetic Phylloseptin-PTa and Phylloseptin-PHa

**Figure 5:**
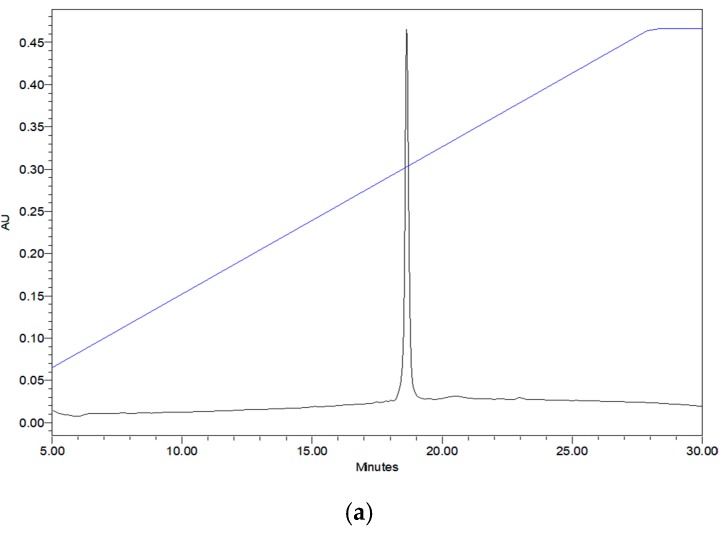

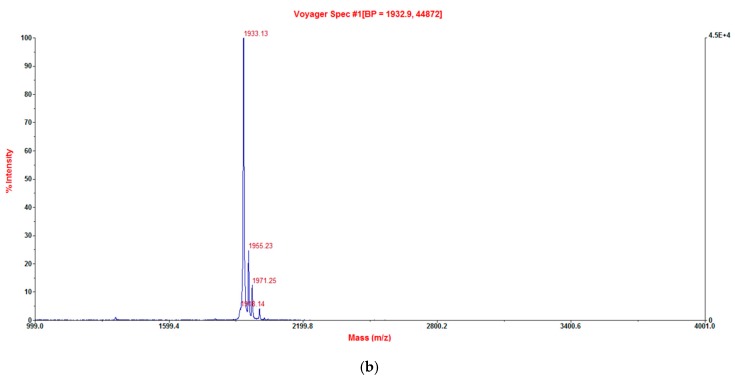
The (**a**) RP-HPLC chromatogram and (**b**) MALDI-TOF MS spectrum of the purified synthetic phylloseptin-PTa.

**Figure 6:**
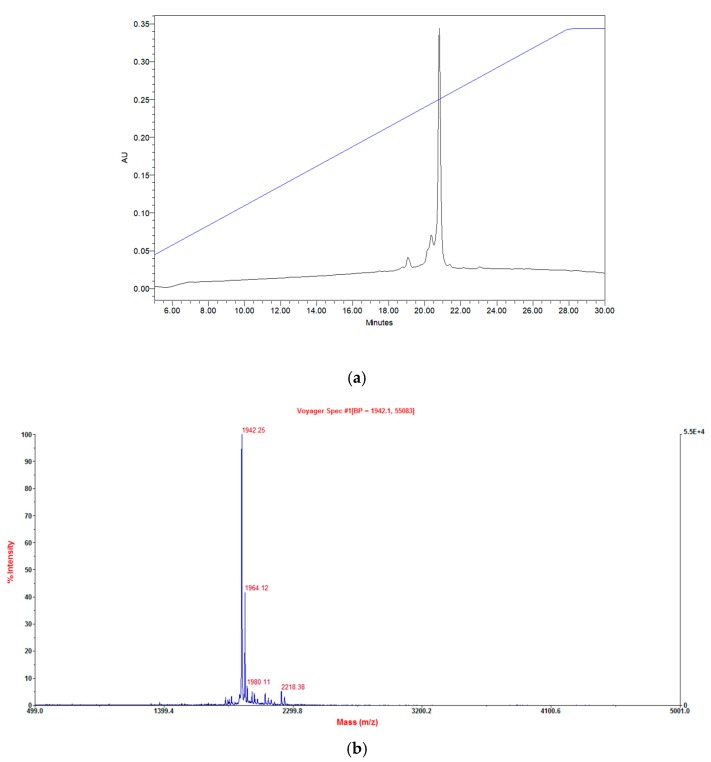
The (**a**) RP-HPLC chromatogram and (**b**) MALDI-TOF mass spectrum of the purified synthetic phylloseptin-PHa.

Phylloseptin-PTa (FLSLIPKIAGGIAALAKHL-NH_2_) and phylloseptin-PHa (FLSLIPAAISAVS ALANHF-NH_2_) were chemically synthesised, purified, and lyophilised prior to being analysed in bioassays. The authenticity of the purified synthetic phylloseptin-PTa and phylloseptin-PHa was verified by RP-HPLC and MALDI-TOF MS, as shown in Figure 5 and Figure 6, respectively, indicating the purity of the purified synthetic peptides no less than 95%.

### 2.4. Secondary Structure Determination of Phylloseptin-PTa and Phylloseptin-PHa by Circular Dichroism

**Figure 7:**
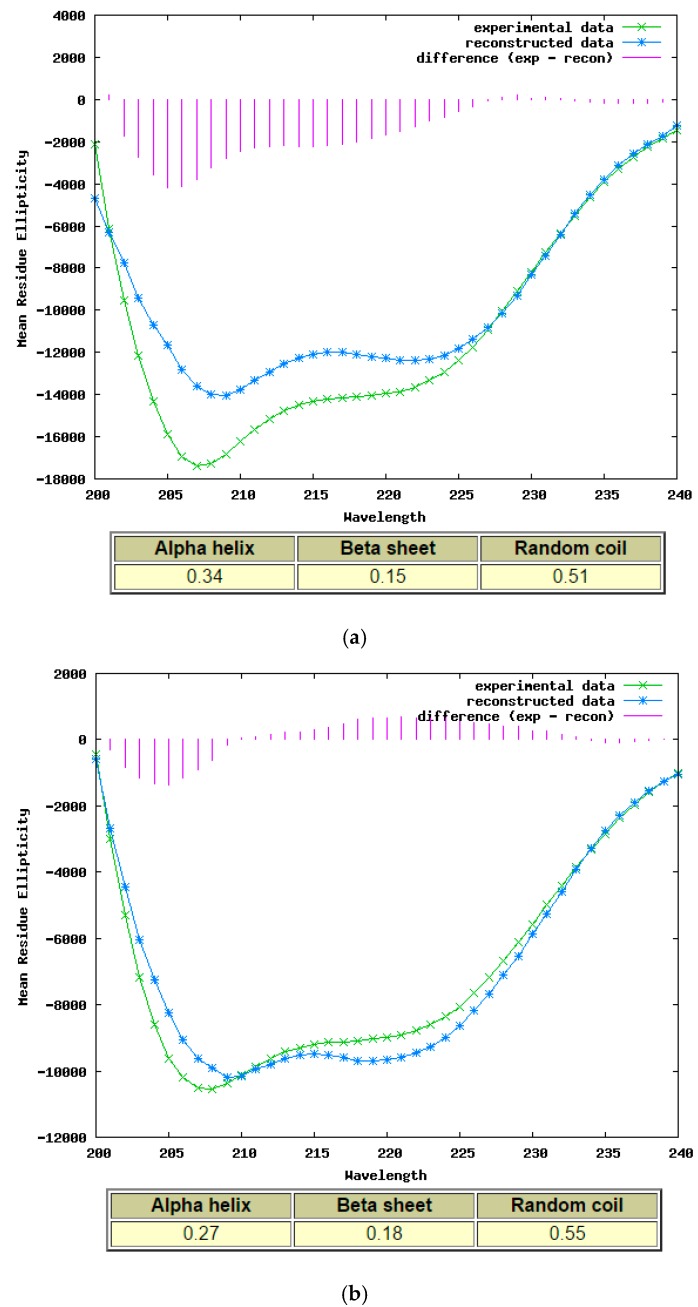
The CD spectra of purified synthetic (**a**) phylloseptin-PTa and (**b**) phylloseptin-PHa plotted as mean residue ellipticity (deg cm^2^ dmol^−1^) versus wavelength (nm) suggested α-helical conformation in the 10 mM NH_4_Ac with 50% TFE analysed from 240 nm to 200 nm using K2D analysis programme and processed via DICHROWEB online server.

The processed spectra in Figure 7 suggest that both purified synthetic phylloseptin-PTa and phylloseptin-PHa displayed α-helical conformation based on double-negative bands at 208 nm and 222 nm in the 10 mM NH_4_Ac with 50% TFE, and the calculated α-helicity was 0.34 and 0.27, respectively, using the K2D analysis programme on the DICHROWEB online server.

### 2.5. Antimicrobial and Haemolytic Activities of Synthetic Phylloseptin-PTa and Phylloseptin-PHa

**Figure 8:**
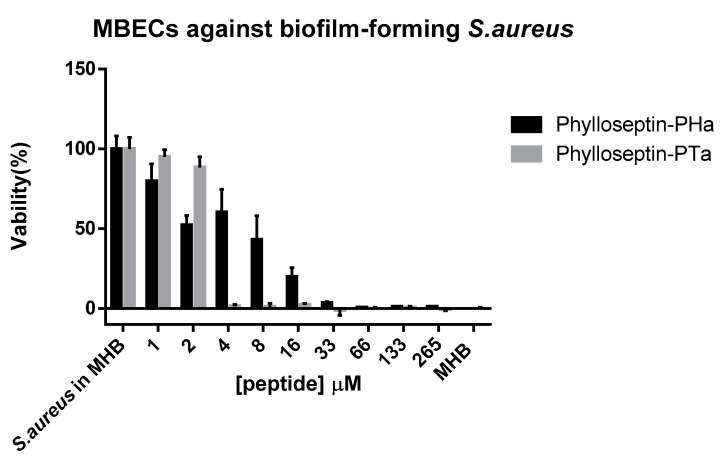
Minimum biofilm eradication concentrations (MBECs) of purified synthetic phylloseptin-PTa and phylloseptin-PHa against biofilm formed by *S. aureus*. Data represent means ± standard error of mean (SEM) of three independent experiments with five replicates.

Two purified synthetic phylloseptins exhibited different strengths of activities against the growth of tested microorganism and horse erythrocytes (Table 2). Phylloseptin-PTa demonstrated a more potent antimicrobial activity than phylloseptin-PHa against all the tested microorganisms and a significant increase of cytotoxicity on erythrocytes. Both synthetic peptides were more potent against tested *Staphylococcus spp.* and *C. albicans* than *E. faecalis* and *E. coli*. Additionally, both peptides were able to eradicate the biofilm formed by *S. aureus* (Figure 8) with phylloseptin-PTa being 8-fold more effective than phylloseptin-PHa. The minimum biofilm eradication concentration (MBEC) of either phylloseptin-PTa or phylloseptin-PHa against *S. aureus* biofilm was identical to their respective MIC recorded for planktonic *S. aureus*.

### 2.6. Scanning Electron Microscopy Observation of S. aureus Biofilms and Peptide Treated S. aureus Biofilms

**Figure 9:**
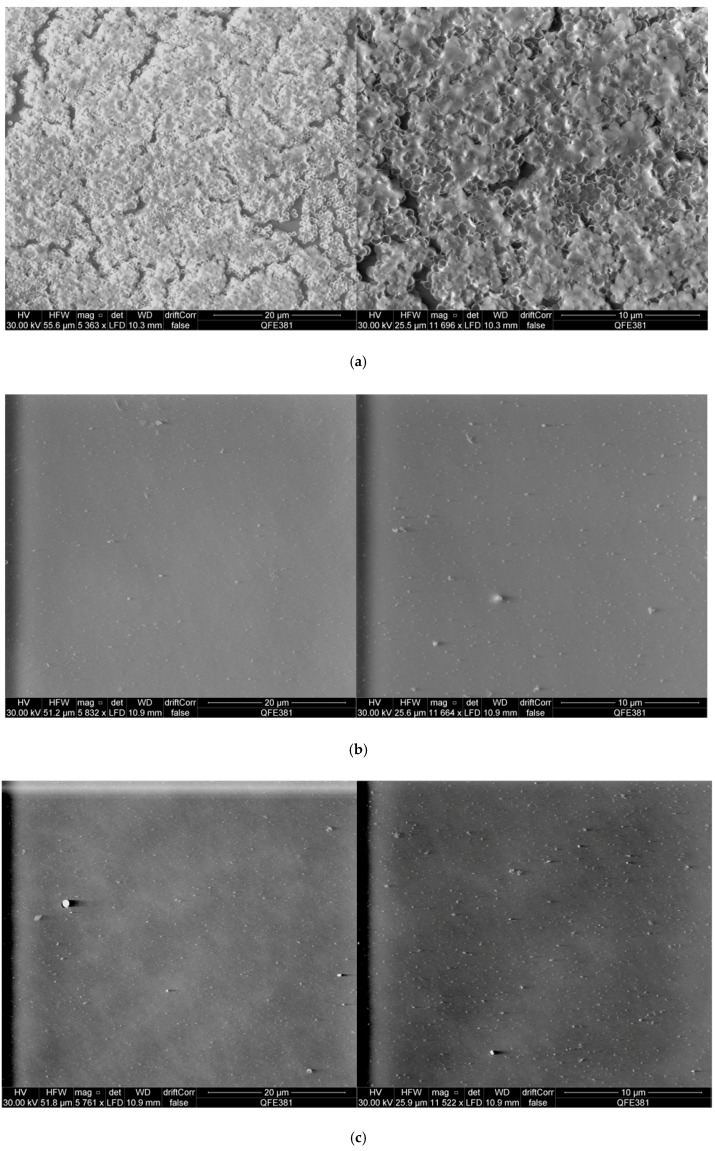

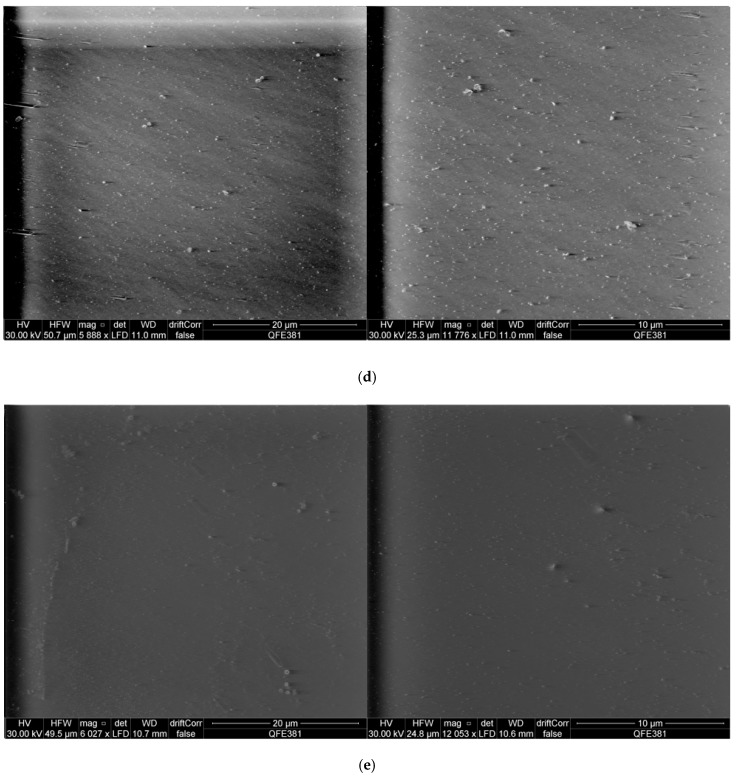
Scanning electron microscopy images of *S. aureus* biofilms treated with purified synthetic phylloseptin-PTa and phylloseptin-PHa. (**a**) Images of *S. aureus* biofilms only; (**b**) Images of *S. aureus* biofilms treated with phylloseptin-PTa at MBEC; (**c**) Images of *S. aureus* biofilms treated with phylloseptin-PTa at 2 × MBEC; (**d**) Images of *S. aureus* biofilms treated with phylloseptin-PHa at MBEC; (**e**) Images of *S. aureus* biofilms treated with phylloseptin-PHa at 2 × MBEC. Scale bars were set at 20 μm and 10 μm.

In order to gain a more informative insight into the interaction of purified synthetic phylloseptin-PTa and phylloseptin-PHa with *S. aureus* biofilms, scanning electron microscopy was employed to observe the untreated *S. aureus* biofilms (Figure 9a) as well as those treated by either of the purified synthetic peptides (Figure 9b–e). The results revealed that both phyllospetins were capable to eradicate the formed biofilm at the concentrations of their respective MBEC and 2 × MBEC.

### 2.7. Cytostatic Activities of Synthetic Phylloseptin-PTa and Phylloseptin-PHa on Non-Small Cell Lung Cancer Cells H157

**Figure 10:**
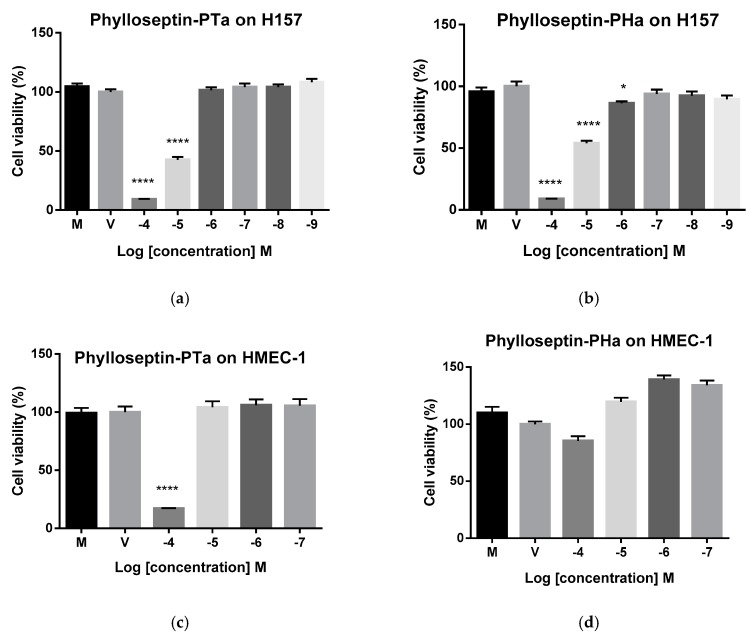
Anti-proliferative effects of purified synthetic (**a**) phylloseptin-PTa and (**b**) phylloseptin-PHa on non-small cell lung cancer cells H157, and purified synthetic (**c**) phylloseptin-PTa and (**d**) phylloseptin-PHa on human microvessel endothelial cells HMEC-1 after 24 h incubation. Data represent means ± SEM of three independent experiments with five replicates. M represents cells in medium, V represents cells in 1% DMSO medium as the vehicle control. **** *p* < 0.0001, * *p* < 0.05 by one-way analysis of variance followed by Dunnett’s multiple comparisons tests with vehicle control group.

Purified synthetic phylloseptin-PTa and phylloseptin-PHa showed cytostatic effects against the proliferation of H157 cells (Figure 10a,b) with the half inhibitory concentration (IC_50_) values of 6.73 μM and 14.10 μM, respectively, calculated by the GraphPad Prism Software (version 6.0). In the meantime, although phylloseptin-PHa exhibited mild cytotoxicity against human microvessel endothelial cells, HMEC-1, phylloseptin-PTa showed considerably cytotoxicity at 100 μM (Figure 10c,d).

### 2.8. Therapeutic Index of Phylloseptin-PTa and Phylloseptin-PHa

The therapeutic indexes (TIs) of phylloseptin-PTa and phylloseptin-PHa in treating Gram-positive bacteria were calculated by comparing HC_10_ to the geometric mean (GM) of MICs of the assessed Gram-positive bacteria [23]_._ The TI for eradicating *S. aureus* forming biofilm was calculated by comparing HC_10_ to MBEC [24]. As shown in Table 3, neither phylloseptin-PTa nor phylloseptin-PHa caused haemolysis at their MICs and MBECs.

## 3. Discussion

The increase in antibiotic resistance incidents worldwide poses a severe threat to the fight against infectious diseases, urging the development of alternative antimicrobial drugs with low resistance induction potential. In this study, two novel phylloseptin peptides were discovered and showed promising antimicrobial activities against the tested planktonic microorganisms with a higher potency against the Gram-positive bacteria including the methicillin-resistant bacteria MRSA. The difference in the antimicrobial activities of phylloseptins on different microorganisms may be a result of the different structure in the target cell membranes. Sheltered under an additional outer membrane with abundant acidic lipopolysaccharides exterior to the inner anionic phospholipid membrane, Gram-negative bacteria are usually more resistant to antibiotics than Gram-positive bacteria, due to their more complex cellular structure.

The classical models of membrane disruption of antimicrobial peptides include the toroidal pore model, carpet model and barrel-stave model, in which peptide-lipid alternating channels, micellar structures and peptide channels, are formed, respectively [5]. Since the MICs of phylloseptin-PTa were relatively low, the mechanism of phylloseptin-PTa was not estimated to be via the carpet model, which is most likely to occur at high peptide concentrations in vitro [5]. Besides, it was reported that the barrel-stave model had only been evidenced for a small number of peptides due to the stringent criteria for forming transmembrane pores. For example, hydrophobic and charged residues should be spaced accurately, and the peptide should be long enough to cross the membrane [25]. Hence, it is speculated that phylloseptin-PTa might act via the toroidal pore model for microbial membrane disruption, which is a well-characterised model deduced mainly from peptides having α-helical secondary structures, such as the magainins, as both phylloseptin-PTa and phylloseptin-PHa formed α-helical secondary structure in a membrane mimic environment. In the toroidal pore model, peptides adopt α-helical structures as they interact with bacterial membranes [4], and by hydrophobic interaction, the hydrophobic areas of peptides interact with the polar head groups of membranes and induce membrane curvature, promoting the bending of the lipid monolayers [4,26]. Similarly, phylloseptin-PHa was also predicted to induce the toroidal pore model to disrupt membrane due to the structural characteristics of phylloseptins. However, the MICs were not as low due to a deficiency of electrostatic interaction, which may decrease the efficiency of the initiating attachment, as well as the large α-helix domain of phylloseptin-PTa than phylloseptin-PHa.

The next interesting character of phylloseptin-PTa and phylloseptin-PHa is their ability to inhibit bacteria biofilms. Biofilms are communities of bacteria embedded in a self-produced matrix, consisting of extracellular polymeric substances including polysaccharides, proteins and DNA. Because the biofilm-forming bacteria are more resistant to current antibiotics and host immune responses, bacterial biofilms often cause chronic and recurrent infections [27,28]. In this light, new treatment methods for biofilm-related infections are required. In order to study the anti-biofilm activity of phylloseptin-PTa and phylloseptin-PHa, an MBEC assay against a *S. aureus-*formed biofilm was carried out. The MBECs of either phylloseptin-PTa or phylloseptin-PHa against *S. aureus* biofilm was identical to their respective MICs recorded for planktonic *S. aureus*. Although the anti-biofilm mechanisms of peptides were not investigated, the identical MIC and MBEC values of each peptide suggest that the mechanisms of anti-biofilm activity might be similar to that involved in killing planktonic bacteria [27]. However, because phylloseptin-PTa showed more potent against *S. aureus* biofilm than phylloseptin-PHa did, it was suggested that the anti-biofilm activity could be influenced by the physiochemical properties of the peptides, such as charges, hydrophobicity and α-helicity.

In addition to their antimicrobial activities, phylloseptin-PTa and phylloseptin-PHa were found to inhibit the proliferation of the non-small lung cancer cell line H157. Previous research has indicated that cancer cell death is correlated with cytomembrane lysis [29]. Since cancer cell membranes are negatively charged, the more positively charged phylloseptin-PTa could better move into contact with the cell surfaces, interact with zwitterionic phospholipids, and lead to more significant membrane disruption and cell death eventually [30,31]. However, both phylloseptin-PTa and phylloseptin-PHa were not able to inhibit the prostate and breast cancer cells, which is likely caused by the high ratio of cholesterol possession of these cells that protect the membrane from interaction with and lysis by the peptides [32].

The superiority of phylloseptin-PTa over the recently discovered phylloseptins include stronger antimicrobial potency and broader antimicrobial spectrum, as shown in Table 4 [15,16,21]. Furthermore, the toxicity of phylloseptin-PTa and phylloseptin-PHa was evaluated using normal mammalian cells and horse red blood cells. Both peptides showed very low toxicity at their effective concentrations in the inhibition of Gram-positive bacteria, *S. aureus*-formed biofilms and non-small cell lung cancer cell line H157, which were collectively reflected by the TI values as shown in Table 3. The selective activities of phylloseptin-PTa and phylloseptin-PHa on different cells are associated with both the characteristics of peptides per se and the target cell membranes. The positively charged amino acid residues of phylloseptin-PTa were suggested to selectively and electrostatically interact with numerous negatively charged molecules, such as phosphatidylserine (PS) and CL in bacteria, PS and *O*-glycosylated mucins in cancer cells, driving phylloseptin-PTa to accumulate around the cell surfaces [33]. As a consequence, the hydrophobicity of phylloseptin-PTa and phylloseptin-PHa could then induce membrane permeabilisation and disruption. The ideal ratios of hydrophobicity of both phylloseptins would produce a lower chance of hydrophobic interaction with the neutral components such as phosphatidylcholine (PC) and phosphatidylethanolamine (PE), in normal cell membranes [4].

## 4. Materials and Methods

### 4.1. Acquisition and Collection of Skin Secretions of P. tarsius and P. hypochondralis

Specimens of adult *P. tarsius* and *P. hypochondrialis* (n = 3) were commercially purchased from Mr. Juan Chavez, Venom Peru Company (PeruBiotech E.I.R.L, Huánuco, Peru) and were maintained in a simulated living environment. These frogs were fed crickets three times per week. After adapting to new surroundings for more than three months, skin secretions were collected from dorsal skin by gentle squeezing [34]. Then the skin secretions were rinsed from the skin with deionised water into a chilled beaker, and snap-frozen in liquid nitrogen and lyophilised. The product was stored at −20 °C prior to analysis. All procedures were vetted and carried out under appropriate UK Animal Research licenses and were consistent with both local and national animal experimentation ethics.

### 4.2. ‘Shotgun’ Cloning of Prepropeptide Encoding cDNAs

An aliquot of 5 mg of lyophilised skin secretions were dissolved in 1 mL of lysis/binding buffer (Life Technologies, Oslo, Norway). Magnetic oligo-dT Dynabeads (Life Technologies) were applied to isolate mRNAs based on A-T pairing according to the manufacturer’s protocol. Then the isolated mRNAs were used as templated for the construction of a cDNA library of *P. tarsius* and *P. hypochondrialis* skin secretion. The cDNA library was subsequently subjected to a 3′-RACE PCR procedures using BD SMART™ RACE cDNA Amplification Kit (Clontech, Palo Alto, CA, USA). The degenerate sense primer (S1; 5′-ACTTTCYGAWTTRYAAGMCCAAABATG-3′) was designed to the highly-conserved domain of the 5′-untranslated region of previously-characterised homologous peptide cDNAs from several species of the *Phyllomedusa* genus, and was used in combination with a nested universal primer (NUP) provided with the kit. The PCR products were purified and cloned using a pGEM-T easy vector system (Promega Corporation, Southampton, UK) and sequenced using an ABI 3730 automated sequencer (Applied Biosystems, Foster City, CA, USA). The similarity of the nucleotide and translated amino acid sequences of the novel peptide precursor with known sequences were analysed using NCBI protein BLAST tool. The sequence alignments were established by the Vector NTI Advance 11.5.1 package. The nucleotide sequence of the cDNA encoding the novel peptide precursor was registered with and is permanently available at the European Nucleotide Archive (ENA) browser at http://www.ebi.ac.uk/ena/data/view/.

### 4.3. Isolation and Identification of Novel Peptides and their Primary Structural Analysis

An aliquot of 5 mg of lyophilised skin secretions were dissolved in 1 mL of trifluoroacetic acid (TFA)/water (0.05/99.95, *v*/*v*) and clarified by centrifugation (2500× *g* for 5 min). The supernatant was then injected and pumped onto a RP-HPLC column (Jupiter, C5, 300 Å, 5 μm, 250 mm × 4.6 mm, Phenomenex, Macclesfield, Cheshire, UK) which was attached to a RP-HPLC system (Waters, Milford, MA, USA). The sample was eluted with a linear gradient from TFA/water (0.05/99.95; *v*/*v*) to TFA/water/acetonitrile (0.05/19.95/80.00; *v*/*v*/*v*) in 240 min at a flow rate of 1 mL/min, and all fractions were collected at one minute intervals. The collected fractions were analysed by a Voyager DE MALDI-TOF MS (Perseptive Biosystems, Bedford, MA, USA) in a positive detection mode using α-cyano-4-hydroxycinnamic acid matrix. The fraction containing the peptide with a molecular mass similar to the putative cDNA-encoding peptide was analysed by tandem mass (MS/MS) fragmentation using an LCQ-Fleet ion-trap mass spectrometer (ThermoFisher Scientific, San Francisco, CA, USA).

### 4.4. Solid-phase Peptide Synthesis of Novel Peptides

Phylloseptin-PTa and phylloseptin-PHa were synthesised by Fmoc solid-phase synthesis using a PS4 Tribute peptide synthesiser (Protein Technologies, Tucson, AZ, USA). Deprotection of the Fmoc groups from the amino acids was performed in 20% piperidine in dimethylformamide (DMF). The coupling of peptide bonds was performed in 11% *N*-methylmorpholine (NMM) in DMF. After all the synthesis cycles were finished, the peptide was cleaved from the Rink amide MBHA resin using 94% TFA, 2% 1,2-ethanedithiol (EDT), 2% thioanisole (TIS) and 2% water at room temperature for 4 h. Next, the cleavage mixture was concentrated and the peptide was precipitated by ice-cold diethyl ether. Afterwards, the precipitated peptide was washed by ice-cold diethyl ether and dissolved in 20 mL of TFA/water (0.05/99.95, *v*/*v*) for lyophilisation.

The synthetic phylloseptin-PTa and phylloseptin-PHa were purified by RP-HPLC and lyophilised prior being analysed in bioassays. The authenticity of the purified synthetic phylloseptin-PTa and phylloseptin-PHa was verified by a Waters RP-HPLC system with an analytical RP-HPLC column (Vydac, C18, 300 Å, 5 μm, 100 mm × 4.6 mm, Hichrom, Berkshire, UK) and MALDI-TOF MS on a linear time-of-flight Voyager DE mass spectrometer (Voyager DE, Perspective Biosystems) in a positive detection mode using α-cyano-4-hydroxycinnamic acid matrix.

### 4.5. Secondary Structure Determination of Novel Peptides by Circular Dichroism

CD spectra were obtained at 20 °C using a 1 mm high precision quartz cell (Hellma Analytics, Essex, UK) with a JASCO J-815 CD spectrometer (Jasco, Essex, UK). The measurement range was from 250 nm to 190 nm at a scanning speed of 200 nm/min. The bandwidth was 1 nm, and the data pitch was 0.5 nm. Phylloseptin-PTa and phylloseptin-PHa were respectively dissolved in 10 mM NH_4_Ac or 10 mM NH_4_Ac with 50% TFE to reach a final concentration of 100 μM. The experimental data were processed via DICHROWEB [35,36,37] and the α-helicity was calculated by K2D analysis programme [38].

### 4.6. Antimicrobial Susceptibility Assays of Novel Peptides

The antimicrobial activities of phylloseptin-PTa and phylloseptin-PHa were evaluated by the determination of MIC values against a panel of microorganisms—the Gram-positive bacteria *S. aureus* (NCTC 10788), *E. faecalis* (NCTC12697), and MRSA (NCTC 12493); the Gram-negative bacterium *E. coli* (NCTC 10418) and the yeast *C. albicans* (NCYC 1467). The microorganisms were cultured to their log phase in Muller-Hinton Broth (MHB), reconstituted to 5 × 10^5^ CFU/mL, and subsequently treated with a series dilution of the peptides. After incubation at 37 °C overnight, the growth of microorganisms was detected at a wavelength of 550 nm using a Synergy HT plate reader (EL808, Biolise BioTek, Winooski, VT, USA). The MICs were determined as the lowest concentration of peptide where no growth was detectable.

### 4.7. Anti-Biofilm Activity using S. aureus Biofilm

Determination of the MBEC of phylloseptin-PTa and phylloseptin-PHa was performed using the manual MBEC P&G assay (Innovotech, Edmonton, AB, Canada). In detail, the log-phase *S. aureus* culture was diluted to 10^7^ CFU/mL and introduced to form biofilms on the surface of the designed pegs for 72 h. The biofilms formed on the pegs were washed twice by PBS and transferred to a challenge plate containing 200 μL of peptide solutions at a concentration series ranging from 512 mg/L to 1 mg/L in doubling dilution. Following incubation at 37 °C for 18 h, the pegs were rinsed twice by PBS and placed in a recovery plate containing 200 μL of 0.5% Tween-80 in MHB. The recovery plate was subsequently sonicated for 15 min and further incubated for 10 h. The absorbance of the recovery plate at a wavelength of 550 nm was detected using a Synergy HT plate reader (Biolise BioTek EL808). The MBEC was determined as the lowest concentration of peptide at which no bacterial growth was detectable.

### 4.8. Scanning Electron Microscopy Observation of Complete S. aureus Biofilms and S. aureus Biofilms Treated with Phylloseptin-PTa and Phylloseptin-PHa

The pegs were broken and fixed in 2.5% glutaraldehyde solution at 4 °C overnight. The fixed biofilms on the pegs were then dehydrated with gradient ethanol 50%, 70%, 90%, 100%, and further air-dried completely. Scanning electron microscopy for the observation of biofilms was performed with a Quanta FEG 250 (ThermoFisher Scientific, San Francisco, CA, USA) an acceleration voltage of 30 kV under environmental mode (0.9 mbar) with standard SEM copper tape as background.

### 4.9. Anticancer Activity Assays of Phylloseptin-PTa and Phylloseptin-PHa

The anticancer activities of phylloseptin-PTa and phylloseptin-PHa against human cells were evaluated by MTT cell viability assays. H157 non-small cell lung cancer cells were cultured in RPMI 1640 medium (Life Technology, Paisley, UK) supplemented with 10% fetal bovine serum (FBS) (Sigma-Aldrich, St. Louis, MO, USA) and 1% penicillin-streptomycin (100 Unit/mL, 100 μg/mL) (Sigma-Aldrich). Cells were cultured in flasks at 37 °C under 5% CO_2_.

The cells were seeded into 96-well plates (5000 cells/well) and cultured for 24 h, after which, the cells were starved for 6 h. A peptide stock solution was prepared using DMSO to achieve 10^−2^ M, and working solutions were diluted from it in medium without FBS. Cells were challenged with peptide solutions at a series of concentrations ranging from 10^−9^ M to 10^−4^ M and incubated for 24 h. Then each well received 10 μL of MTT solution (5 mg/mL, Thermo Fisher Scientific). Cells were cultured for a further 4 h, after which the supernatants were removed from wells and 100 μL of DMSO were added into all wells. After gentle shaking to dissolve the formazan crystals, the absorbance was detected at a wavelength of 570 nm using a Synergy HT plate reader (Biolise BioTek EL808). The cell viabilities at different concentrations of peptide solution were calculated as follows:

Viability%=A∕A0×100%

where A represents the average absorbance of sample groups at the same concentrations. A0 represents the average absorbance of vehicle controls (1% DMSO in medium).

The IC_50_ against different cell lines were determined using GraphPad Prism software (Version 6.0; GraphPad Software Inc., San Diego, CA, USA). Results were presented as mean ± SEM, and data were statistically analysed using one-way analysis of variance followed by Dunnett’s multiple comparisons tests with vehicle control group in GraphPad Prism Software (Version 6.0). Groups of data were considered to be significantly different if the *P* value was less than 0.05.

### 4.10. Cytotoxicity Evaluations of Phylloseptin-PTa and Phylloseptin-PHa

The cytotoxicities of phylloseptin-PTa and phylloseptin-PHa were measured on HMEC-1 cells using MTT cell viability assay, as described above. Specifically, HMEC-1 cells were grown in MCDB131 medium (Life Technology) with added 10% FBS, 10 ng/mL epidermal growth factor (EGF) (Life Technology), 10 mM L-Glutamine (Life Technology) and 1% penicillin-streptomycin (Sigma-Aldrich). Results were presented as mean ± SEM, and values were compared using one-way analysis of variance followed by Dunnett’s multiple comparisons tests with vehicle group in GraphPad Prism Software (Version 6.0). Groups of data were considered to be significantly different if the *P* value was less than 0.05.

### 4.11. Haemolysis Evaluations of Phylloseptin-PTa and Phylloseptin-PHa

A 4% (*v*/*v*) suspension of erythrocytes was prepared from defibrinated horse blood (TCS Biosciences Ltd., Buckingham, UK) by washing and centrifugation for several times. Equal volumes (200 μL) of peptide solutions and erythrocyte suspensions were incubated together at 37 °C for 2 h. Lysis of erythrocytes was detected by the measurement of supernatant absorbance at a wavelength of 550 nm using a Synergy HT plate reader (Biolise BioTek EL808). The negative controls consisted of equal volumes of erythrocyte suspension and PBS and positive control consisted of equal volumes of erythrocyte suspension and 2% Triton X-100 (Sigma-Aldrich) in PBS solution while the vehicle controls consisted of equal volumes of erythrocyte suspension and 1% DMSO in PBS solution. The equation for the calculation of the haemolytic rate was shown below:

Haemolysis%=(A−A0)∕(A1−A0)×100%,

where A represents the average absorbance with peptide treatments, A0 represents the average absorbance in the vehicle control group and A1 represents the average absorbance in the positive control group. The HC_50_ value and HC_10_ value were determined by GraphPad Prism software (Version 6.0).

## 5. Conclusions

In conclusion, the naturally selected AMPs, phylloseptin-PTa, identified from the skin secretion of *P. tarsius*, and phylloseptin-PHa, identified from the skin secretion of *P. hypochondrialis*, showed effective antimicrobial activities on the tested Gram-positive bacteria and the antibiotic-resistant bacteria MRSA, and displayed strong biofilm eradicative activity while inhibiting the proliferation of non-small cell lung cancer cells H157. Interestingly, phylloseptin-PTa demonstrated better potency than phylloseptin-PHa in all the models that were examined. Therefore, phylloseptin-PTa could be utilised as an antimicrobial lead to provide new ideas for the development of novel antimicrobial therapeutics and deserves further comprehensive study.

## Figures and Tables

**Table 1:** Predicted singly-charged *b* ions and *y* ions arising from MS/MS fragmentation of (**a**) phylloseptin-PTa and (**b**) phylloseptin-PHa. Actual fragment ions observed following MS/MS fragmentation are indicated in blue and red.

#1	*b*(1+)	*b*(2+)	Seq.	*y*(1+)	*y*(2+)	#2
**(a)**						
1	148.08	74.54	F			19
2	261.16	131.08	L	1785.15	893.08	18
3	348.19	174.60	S	1672.06	836.54	17
4	461.15	231.14	L	1585.03	793.02	16
5	574.18	287.68	I	1471.95	736.48	15
6	671.69	336.21	P	1358.75	679.94	14
7	799.51	400.26	K	1261.81	631.41	13
8	912.59	456.80	I	1133.71	567.36	12
9	983.63	492.32	A	1020.71	510.82	11
10	1040.65	520.83	G	949.59	475.30	10
11	1097.67	549.34	G	892.82	446.79	9
12	1210.71	605.88	I	835.55	418.28	8
13	1281.79	641.40	A	722.47	361.74	7
14	1352.83	676.92	A	651.43	326.22	6
15	1465.82	733.46	L	580.39	290.70	5
16	1536.94	768.98	A	467.31	234.16	4
17	1664.89	833.03	K	396.27	198.64	3
18	1802.11	901.56	H	268.18	134.59	2
19			L-Amidated	131.12	66.06	1
**(b)**						
1	148.08	74.54	F			19
2	261.16	131.08	L	1794.03	897.52	18
3	348.19	174.60	S	1680.94	840.98	17
4	461.28	231.14	L	1593.91	797.46	16
5	574.17	287.68	I	1480.83	740.92	15
6	671.09	336.21	P	1367.68	684.38	14
7	742.45	371.73	A	1270.69	635.85	13
8	813.49	407.25	A	1199.65	600.33	12
9	926.57	463.79	I	1128.80	564.81	11
10	1013.60	507.31	S	1015.53	508.27	10
11	1084.64	542.82	A	928.50	464.75	9
12	1183.71	592.36	V	857.46	429.24	8
13	1270.74	635.87	S	758.37	379.70	7
14	1341.78	671.39	A	671.36	336.18	6
15	1454.64	727.93	L	600.17	300.67	5
16	1525.67	763.45	A	487.31	244.12	4
17	1640.71	820.47	N	416.19	208.61	3
18	1777.00	889.00	H	302.16	151.58	2
19			F-Amidated	165.10	83.05	1

**Table 2:** Antimicrobial activity-minimum inhibitory concentration (MIC) determinations and haemolytic activity of purified synthetic phylloseptins. The half haemolytic concentration (HC_50_) and 10% haemolytic concentration (HC_10_) were analyse by Graphpad Prism Software (version 6.0).

	MIC (µg∙mL^−1^/ µM)					Haemolysis		
Peptide	*S. aureus* NCTC 10788	MRSA NCTC 12493	*E. faecalis* NCTC 12697	*E. coli* NCTC 10418	*C. albicans* NCYC 1467	% lysis at MIC ^1^	HC_10_ (μM)	HC_50_ (μM)
**Phylloseptin-PTa**	8/4.14	8/4.14	32/16.56	32/16.56	4/2.07	4.42%	7.79	22.8
**Phylloseptin-PHa**	64/32.97	64/32.97	512/263.78	>512/>263.78	256/131.89	2.52%	76.5	109.5

^1^ MIC observed against *S. aureus*.

**Table 3:** TIs of phylloseptin-PTa and phylloseptin-PHa as Gram-positive bacteriostatic agents and *S. aureus* biofilm eradicating agents

	TI on Gram-Positive Bacteria	TI on *S. aureus* Biofilm
Peptide	HC_10_	HC_10_
	/GM	/MBEC
Phylloseptin-PTa	1.19	1.88
Phylloseptin-PHa	1.66	3.32

**Table 4:** The comparisons of phylloseptin-PTa with the recently reported phylloseptins in terms of characteristics and biological activities.

MIC (µg∙mL^−1^/µM)						
	Phylloseptin-PTa	Phylloseptin-PHa	Phylloseptin-Du	Phylloseptin-Co	Phylloseptin-PBa	Phylloseptin-PT
*S. aureus*	8/4.14	64/32.97	8/3.90	8/4.06	8/3.99	512/264.33
*C. albicans*	4/2.07	256/131.89	16/7.81	16/8.12	8/3.99	512/264.33
*E. coli*	32/16.56	>512/>263.78	128/62.45	128/64.93	128/63.84	>512/>264.33
**MBEC (µg∙mL^−1^/µM)**						
*S. aureus*	8/4.14	64/32.97	16/7.81	16/8.12	nr ^3^	nr ^3^

^3^ Means not reported.

## Abbreviations

CL	cardiolipin
EDT	1,2-ethonedithiol
MBEC	minimum biofilm eradication concentration
PC	phosphatidylcholine
PE	phosphatidylethanolamine
PG	phosphatidylglycerol
PS	phosphatidylserine
RP-HPLC	reversed-phase high performance liquid chromatography
TFA	trifluoroacetic acid
TFE	trifluoroethanol
TIS	thioanisole

## References

[1] Toledo R.D., Jared C. (1995). Cutaneous granular glands and amphibian venoms. Comp. Biochem. Physiol. A Physiol..

[2] Pukala T.L., Bowie J.H., Maselli V.M., Musgrave I.F., Tyler M.J. (2006). Host-defence peptides from the glandular secretions of amphibians: structure and activity. Nat. Prod. Rep..

[3] Bowie J.H., Separovic F., Tyler M.J. (2012). Host-defense peptides of Australian anurans. Part 2. Structure, activity, mechanism of action, and evolutionary significance. Peptides.

[4] Yeaman M.R., Yount N.Y. (2003). Mechanisms of antimicrobial peptide action and resistance. Pharmacol. Rev..

[5] Nguyen L.T., Haney E.F., Vogel H.J. (2011). The expanding scope of antimicrobial peptide structures and their modes of action. Trends Biotechnol..

[6] Erspamer V., Melchiorri P., Erspamer G.F., Montecucchi P., De Castiglione R. (1985). Phyllomedusa skin: A huge factory and store-house of a variety of active peptides. Peptides.

[7] Amiche M., Ladram A., Nicolas P. (2008). A consistent nomenclature of antimicrobial peptides isolated from frogs of the subfamily Phyllomedusinae. Peptides.

[8] Pyron R.A., Wiens J.J. (2011). A large-scale phylogeny of Amphibia including over 2800 species, and a revised classification of extant frogs, salamanders, and caecilians. Mol. Phylogenet. Evol..

[9] Jackway R.J., Pukala T.L., Donnellan S.C., Sherman P.J., Tyler M.J., Bowie J.H. (2011). Skin peptide and cDNA profiling of Australian anurans: Genus and species identification and evolutionary trends. Peptides.

[10] König E., Bininda-Emonds O.R., Shaw C. (2015). The diversity and evolution of anuran skin peptides. Peptides.

[11] Vanhoye D., Bruston F., Nicolas P., Amiche M. (2003). Antimicrobial peptides from hylid and ranin frogs originated from a 150-million-year-old ancestral precursor with a conserved signal peptide but a hypermutable antimicrobial domain. Eur. J. Biochem..

[12] de Azevedo Calderon L., Alexandre de Almeida E.S., Ciancaglini P., Stábeli R.G. (2011). Antimicrobial peptides from Phyllomedusa frogs: From biomolecular diversity to potential nanotechnologic medical applications. Amino Acids.

[13] Mura M., Wang J., Zhou Y., Pinna M., Zvelindovsky A.V., Dennison S.R., Phoenix D.A. (2016). The effect of amidation on the behaviour of antimicrobial peptides. Eur. Biophys. J..

[14] Pan Y., Wan J., Roginski H., Lee A., Shiell B., Michalski W., Coventry M. (2007). Comparison of the effects of acylation and amidation on the antimicrobial and antiviral properties of lactoferrin. Lett. Appl. Microbiol..

[15] Wan Y., Ma C., Zhou M., Xi X., Li L., Wu D., Wang L., Lin C., Lopez J.C., Chen T. (2015). Phylloseptin-PBa—A novel broad-spectrum antimicrobial peptide from the skin secretion of the peruvian purple-sided leaf frog (phyllomedusa baltea) which exhibits cancer cell cytotoxicity. Toxins (Basel).

[16] Yang N., Li L., Wu D., Gao Y., Xi X., Zhou M., Wang L., Chen T., Shaw C. (2016). Discovery of novel bacterial cell-penetrating phylloseptins in defensive skin secretions of the south american hylid frogs, phyllomedusa duellmani and phyllomedusa coelestis. Toxins (Basel).

[17] Zhang R., Zhou M., Wang L., McGrath S., Chen T., Chen X., Shaw C. (2010). Phylloseptin-1 (PSN-1) from phyllomedusa sauvagei skin secretion: A novel broad-spectrum antimicrobial peptide with antibiofilm activity. Mol. Immunol..

[18] Leite J.R.S., Silva L.P., Rodrigues M.I.S., Prates M.V., Brand G.D., Lacava B.M., Azevedo R.B., Bocca A.L., Albuquerque S., Bloch C. (2005). Phylloseptins: A novel class of anti-bacterial and anti-protozoan peptides from the Phyllomedusa genus. Peptides.

[19] Raja Z., André S., Piesse C., Sereno D., Nicolas P., Foulon T., Oury B., Ladram A. (2013). Correction: Structure, antimicrobial activities and mode of interaction with membranes of bovel phylloseptins from the painted-belly leaf frog, Phyllomedusa sauvagii. PLoS ONE.

[20] Pinto E.G., Pimenta D.C., Antoniazzi M.M., Jared C., Tempone A.G. (2013). Antimicrobial peptides isolated from Phyllomedusa nordestina (Amphibia) alter the permeability of plasma membrane of Leishmania and Trypanosoma cruzi. Exp. Parasitol..

[21] Gao Y., Wu D., Xi X., Wu Y., Ma C., Zhou M., Wang L., Yang M., Chen T., Shaw C. (2016). Identification and characterisation of the Antimicrobial Peptide, Phylloseptin-PT, from the Skin Secretion of Phyllomedusa tarsius, and Comparison of Activity with Designed, Cationicity-Enhanced Analogues and Diastereomers. Molecules.

[22] Chen T., Zhou M., Gagliardo R., Walker B., Shaw C. (2006). Elements of the granular gland peptidome and transcriptome persist in air-dried skin of the South American orange-legged leaf frog, Phyllomedusa hypocondrialis. Peptides.

[23] Jacob B., Rajasekaran G., Kim E.Y., Park I.-S., Bang J.-K., Shin S.Y. (2016). The stereochemical effect of SMAP-29 and SMAP-18 on bacterial selectivity, membrane interaction and anti-inflammatory activity. Amino Acids.

[24] Mojsoska B., Carretero G., Larsen S., Mateiu R.V., Jenssen H. (2017). Peptoids successfully inhibit the growth of gram negative E. coli causing substantial membrane damage. Sci. Rep..

[25] Schmidtchen A., Pasupuleti M., Malmsten M. (2014). Effect of hydrophobic modifications in antimicrobial peptides. Adv. Colloid Interface Sci..

[26] Harrison P.L., Heath G.R., Johnson B.R., Abdel-Rahman M.A., Strong P.N., Evans S.D., Miller K. (2016). Phospholipid dependent mechanism of smp24, an α-helical antimicrobial peptide from scorpion venom. Biochim. Biophys. Acta Biomembr..

[27] Batoni G., Maisetta G., Esin S. (2016). Antimicrobial peptides and their interaction with biofilms of medically relevant bacteria. Biochim. Biophys. Acta Biomembr..

[28] Høiby N., Bjarnsholt T., Givskov M., Molin S., Ciofu O. (2010). Antibiotic resistance of bacterial biofilms. Int. J. Antimicrob. Agents.

[29] Papo N., Shai Y. (2005). Host defense peptides as new weapons in cancer treatment. CMLS Cell. Mol. Life Sci..

[30] Gaspar D., Veiga A.S., Castanho M.A. (2013). From antimicrobial to anticancer peptides. A review. Front. Microbiol..

[31] Matsuzaki K. (2009). Control of cell selectivity of antimicrobial peptides. Biochim. Biophys. Acta Biomembr..

[32] Li Y.C., Park M.J., Ye S.-K., Kim C.-W., Kim Y.-N. (2006). Elevated levels of cholesterol-rich lipid rafts in cancer cells are correlated with apoptosis sensitivity induced by cholesterol-depleting agents. Am. J. Pathol..

[33] Schweizer F. (2009). Cationic amphiphilic peptides with cancer-selective toxicity. Eur. J. Pharmacol..

[34] Tyler M.J., Stone D.J., Bowie J.H. (1992). A novel method for the release and collection of dermal, glandular secretions from the skin of frogs. J. Pharmacol. Toxicol. Methods.

[35] Whitmore L., Wallace B.A. (2008). Protein secondary structure analyses from circular dichroism spectroscopy: Methods and reference databases. Biopolymers.

[36] Whitmore L., Wallace B.A. (2004). Dichroweb: An online server for protein secondary structure analyses from circular dichroism spectroscopic data. Nucleic Acids Res..

[37] Lobley A., Whitmore L., Wallace B.A. (2002). Dichroweb: An interactive website for the analysis of protein secondary structure from circular dichroism spectra. Bioinformatics.

[38] Andrade M.A., Chacón P., Merelo J.J., Morán F. (1993). Evaluation of secondary structure of proteins from UV circular dichroism using an unsupervised learning neural network. Protein Eng. Des. Sel..

